# Nutrition, Microbiota and Noncommunicable Diseases

**DOI:** 10.3390/nu12071971

**Published:** 2020-07-02

**Authors:** Julio Plaza-Diaz

**Affiliations:** 1 Department of Biochemistry and Molecular Biology II, School of Pharmacy, University of Granada, 18,071 Granada, Spain; jrplaza@ugr.es; Tel.: +34-958-241-599; 2 Instituto de Investigación Biosanitaria IBS.GRANADA, Complejo Hospitalario Universitario de Granada, 18,014 Granada, Spain; 3 Children’s Hospital of Eastern Ontario Research Institute, Ottawa, ON K1H 8L1, Canada

The advent of new sequencing technologies has inspired the foundation of novel research to ascertain the connections between the microbial communities that reside in our gut and some physiological and pathological conditions. The microbiota, defined as the full collection of microbes (bacteria, fungi, and viruses, among others) that naturally exist within a particular biological niche, is estimated to contain 500–1000 species [1,2,3,4].

This Special Issue of *Nutrients*, “Nutrition, Microbiota, and Noncommunicable Diseases” contains 13 original publications and seven reviews investigating the contribution of intestinal microbiota on relevant health outcomes in a variety of populations, and animal studies which suggest the growing and extensive interests of research on this topic.

Seven studies were published examining the changes in intestinal microbiota in the human population. Two of these studies recruited patients with metabolic syndrome. Tenorio-Jimenez et al. [5] reported the anthropometric variables and biochemical and inflammatory biomarkers as well as the gastrointestinal microbiome composition changes in a randomized, crossover, placebo-controlled, single-center trial in adult patients newly diagnosed with metabolic syndrome treated either with *Lactobacillus reuteri* V3401 or a placebo during 12 weeks. *L. reuteri* V3401 administration improved selected inflammatory parameters and modified the gastrointestinal microbiome, especially *Verrucomicrobia* [5], and Bellikci-Koyu et al. [6] investigated the effects of regular kefir consumption on gut microbiota composition, and their relation with the components of metabolic syndrome in a parallel-group, randomized, controlled clinical trial for 12 weeks. Gut microbiota analysis showed that regular kefir consumption resulted in a significant increase only in *Actinobacteria* abundance [6].

In two more additional studies, one with healthy elderly women and another with patients with non-alcoholic fatty liver disease (NAFLD), Morita et al. [7] examined the effect of an exercise intervention (12 weeks, trunk muscle training or aerobic exercise training) on the composition of the intestinal microbiota in healthy elderly women. *Bacteroides* abundance was significantly increased only in the aerobic exercise group, particularly in subjects showing increases in the time spent in brisk walking [7], and Chong et al. [8] determined whether inulin supplementation after brief metronidazole therapy is effective in reducing alanine aminotransferase and maintaining weight loss achieved through a very-low-calorie diet among people with NAFLD. Treatment decreased the ratio of Firmicutes/Bacteroidetes [8].

Lau et al. [9] evaluated the association of probiotic ingestion with obesity, type 2 diabetes, hypertension, and dyslipidemia using data from the National Health and Nutrition Examination Survey, 1999–2014. Probiotic supplementation or yogurt consumption were associated with a lower prevalence of obesity and hypertension [9]. In another study with humans, Dalla Via et al. [10] verified whether trimethylamine-N-oxide urinary levels may be associated with the fecal relative abundance of specific bacterial taxa and the bacterial choline trimethylamine-lyase gene *cutC* in human fecal samples. Correlation analysis showed that the cut-Kp gene cluster was significantly associated with *Enterobacteriaceae* [10].

Finally, in one study with the pediatric population, Kong et al. [11] reported both oral and intestinal microbiota in patients with autism spectrum disorder and controls, with specific microbial patterns [11].

Regarding animal studies, six studies were published examining the changes in intestinal microbiota. Probiotic supplementation, high-fat diet, use of anorexic mice, fiber, and soy intake and antihypertensive effect in metabolomics profiles were analyzed in these studies. Valcarce et al. [12] reported the effect of a short-time probiotic supplementation consisting of a mixture of two probiotic bacteria with proven antioxidant and anti-inflammatory activities on zebrafish sperm quality and male behavior [12]. Hsu et al. [13] examined the alterations of gut microbiota, mediation of short-chain fatty acids (SCFAs) and their receptors, and downregulation of nutrient-sensing signals effects in rats that received a high-fat diet. Increased Firmicutes to Bacteroidetes ratio, *Akkermansia* and *Verrucomicrobia*, and reduced abundance in the genus *Lactobacillus* were associated with blood pressure elevation [13]. Dominique et al. [14] investigated the role of the microbiome and the ClpB protein in the deregulation and self-maintenance of anorexia pathology in mice. Plasma concentration of ClpB was increased in both limited food access and activity-based anorexia mice and it was correlated with the proportion of Enterobacteriaceae in the animal feces [14]. Sasaki et al. [15] investigated the effects of fiber intake timing on metabolism. Data have suggested that inulin is more easily digested by fecal microbiota during the active period than the inactive period. Inulin consumption at breakfast has a greater effect on the microbiota [15]. Tamura et al. [16] investigated soy protein intake effects on intestinal microbiota. Soy protein intake whether in the morning or evening led to a greater microbiota diversity and a decrease in cecal pH resulting from SCFA production compared with casein intake [16]. Finally, Ahn et al. [17] investigated the metabolomics changes in rats that received amlodipine. Serum levels of phosphatidylcholine, lysophosphatidylcholine, sphingomyelin, triglycerides with large numbers of double bonds, cholesterol, sterol derivatives, and cholesterol esters were increased. Amlodipine-induced compositional changes in the gut microbiota are a causal factor in inflammation [17].

Seven reviews investigating the impact of intestinal microbiota on relevant health outcomes in a variety of populations were published. Hills Jr. et al. [18] described a general vision about the gut microbiome and its important role in human health. Salli et al. [19] reported the health benefits of xylitol. The other reviews have described the intestinal microbiota changes in specific conditions, early infancy, hepatic ischemia-reperfusion and regeneration in liver surgery, vaginal microbiota, and cardiovascular diseases. Mesa et al. [20] reported the microbiome changes and how those modulate the inflammatory mechanisms related to physiological and pathological processes that are involved in the perinatal progress. Cornide-Petronio et al. [21] summarized the role of starvation, supplemented nutrition diet, nutritional status, and alterations in microbiota on hepatic ischemia/reperfusion and regeneration. Barrientos-Duran et al. [22] examined the most important aspect in the vaginal microbiota, with special emphasis in bacterial vaginosis, and the maintenance of eubiosis, and Sanchez-Rodriguez et al. [23] discussed how external factors such as dietary and physical activity habits influence host microbiota and atherogenesis, the potential mechanisms of the influence of gut microbiota in host blood pressure, and the alterations in the prevalence of those bacterial genera affecting vascular tone and the development of hypertension. Finally, Plaza-Diaz et al. [24] revisited the effects of sweeteners on gut microbiota.

The present Special Issue provides a summary of the progress on the topic of intestinal microbiota and its important role in human health in different populations, which will be of interest from a clinical and public health perspective. Nevertheless, more studies with more samples and comparable methods are necessary to understand the actual function of intestinal microbiota in disease development and health maintenance.
